# New perspectives on the models of porous carbon

**DOI:** 10.1016/j.csbj.2025.04.024

**Published:** 2025-04-22

**Authors:** Jose-I. Beltrán-Larrotta, Juan Carlos Moreno-Piraján, Liliana Giraldo

**Affiliations:** a Departamento de Quimica Facultad de Ciencias, Grupo de Investigacion en Sólidos Porosos y Calorimetría, Universidad de Los Andes, Cra. 1a, No. 18A-10, Bogotá, DC 11711, Colombia; b Departamento de Química, Grupo de Calorimetría, Universidad Nacional de Colombia, Sede Bogota Cra.30 No 45- 03, Bogotá, DC 11711, Colombia

**Keywords:** Activated carbon, Models, GCM, MD

## Abstract

Activated carbon is a versatile material widely used in applications such as water and air purification, energy storage, and catalysis. Its unique structure and properties are derived from an intricate process of carbonization and activation, which creates a complex porous network with varied chemical functionality. This article reviews recent advances in the experimental characterization of activated carbons, highlighting techniques such as X-ray diffraction, infrared spectroscopy, and high-resolution electron microscopy, which have enabled a deeper understanding of their microstructure. Additionally, theoretical models, including Grand Canonical Monte Carlo (GCM) simulations and Molecular Dynamics (MD), are discussed as they complement experimental findings and allow for the prediction of properties such as adsorption and pore size distribution. Finally, emerging trends in the design of activated carbon materials are explored, emphasizing their potential for sustainable applications in environmental remediation and advanced energy technologies.

## Highlights

• Monte Carlo simulations and molecular dynamics.
• Sustainable applications in environmental remediation.
• The Role of Computational Techniques in Model Development.
• Quench Molecular Dynamics (QMD) and Reactive State Sum Potentials (RSS).

## Graphical Abstract

**Figure:**
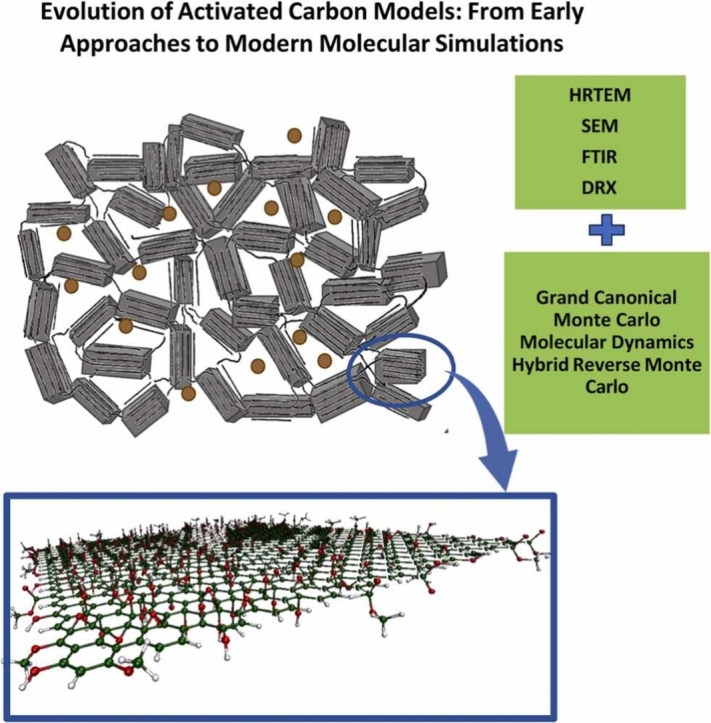
Evolution of Activated Carbon Models: From Early Approaches to Modern Molecular Simulations

## 1 Introduction

Activated carbon (AC) is a porous carbonaceous material with unprecedented relevance in environmental, energy, and industrial applications. Its versatility arises from a unique combination of high specific surface area (500–3000 m²/g), tunable porosity (ranging from micropores <2 nm to mesopores 2–50 nm), and modifiable surface chemistry through the incorporation of oxygen-, nitrogen-, or sulfur-containing functional groups. These properties have positioned AC as a critical adsorbent in water and air purification, an efficient catalyst in redox reactions, and an essential component in energy storage systems such as supercapacitors and batteries. However, its complex hierarchical structure—characterized by a disordered network of curved graphitic fragments, topological defects (e.g., pentagonal/heptagonal rings), and heteroatoms—poses significant challenges for accurate characterization and atomistic modeling. Historically, understanding the microstructure of AC has relied on experimental techniques such as X-ray diffraction (XRD), high-resolution transmission electron microscopy (HRTEM), and Raman spectroscopy. Nevertheless, these methods offer a fragmented perspective, limited by spatial resolution or their inability to capture molecular dynamics in real time. For instance, while HRTEM reveals the turbostratic arrangement of the basic structural units (BSUs) proposed by Oberlin, it fails to elucidate how non-hexagonal defects influence gas adsorption or electronic conductivity.

The advent of advanced computational methods—such as Grand Canonical Monte Carlo (GCMC), Molecular Dynamics (MD), and Hybrid Reverse Monte Carlo (HRMC)—has revolutionized the field. These approaches enable the reconstruction of atomistic models that integrate experimental data (e.g., pore size distributions, adsorption isotherms) with fundamental physicochemical principles. For example, HRMC has facilitated the modeling of anisotropic carbons, such as activated carbon fibers (ACFs), by incorporating long-range interactions and curvature of graphene layers—capabilities unattainable with classical approaches. Moreover, methods such as Quenched Molecular Dynamics (QMD) have shed light on how carbonization cooling rates impact micropore formation and the sp²/sp³ hybridization ratio. This article not only reviews recent advances in the computational modeling of activated carbon but also highlights its synergy with emerging experimental techniques, such as positron annihilation lifetime spectroscopy (PALS) for nanopore mapping and 4D electron microscopy for capturing structural dynamics. These developments are essential for the rational design of "tailor-made" materials optimized to tackle global challenges such as CO₂ capture, hydrogen storage, and the remediation of emerging contaminants.

### 1.1 Formation of activated carbon

Activated carbon is primarily composed of carbon atoms, often interspersed with heteroatoms such as oxygen, nitrogen, or sulfur. The formation process begins with the carbonization of organic precursors, such as wood, coconut shells, or synthetic polymers, followed by an activation step that enhances porosity. Solid carbons include well-known allotropes like graphite and diamond, which serve as benchmarks for modeling more complex structures like activated carbon Rodriguez-Reinoso [48]*;* Marsh and Reinoso [32]*;* Harris [21].

In modeling these structures, deviations from the ideal arrangements of graphite or diamond are crucial Rodriguez- Reinoso [48]; Marsh and Reinoso [32]; Harris [21]. Graphite is characterized by layers of carbon atoms arranged in a hexagonal lattice, where each carbon atom is hybridized sp^2^ and forms sigma bonds with three neighboring carbon atoms, resulting in a bond length of approximately 0.145 nm. The graphene layers are separated by about 0.3354 nm and are held together by delocalized -electrons, contributing to graphite’s notable electrical conductivity and lubricating properties Thomas and Walker [51].

Diamond, conversely, features a three-dimensional tetragonal structure formed by *sp*^2^ hybridized carbon atoms. The two primary polytypes of diamond—cubic and hexagonal—exhibit distinct lattice constants and arrangements Rodriguez-Reinoso [48]*;* Marsh and Reinoso [32]*;* Thomas and Walker [51]*;* Fitzer, Kochling, Boehm and Marsh [15]. Cubic diamond has a lattice constant of approximately 0.3567 nm, while hexagonal diamond has lattice constants of around 0.252 nm and 0.412 nm, respectively. The presence of defects and heteroatoms can significantly influence the properties of diamond, affecting its electronic and optical characteristics Rodriguez-Reinoso [48]*;* Marsh and Reinoso [32]*;* Thomas and Walker [51]*.*

However, not all carbon materials subjected to carbonization achieve a crystalline graphite structure, particularly at high temperatures (exceeding 3000 °C). Those that do not are classified as non-graphitizing carbons, which possess unique properties such as high hardness, low density, and a tendency toward isotropic structures with microporosity Franklin [17]*;* McEnaney [33]*;* Edwards, Marsh and Menendez [12]*;* Yang [56]*;* Patrick [45]*;* Foley [16]. This diversity in carbon structure necessitates advanced modeling techniques to capture the complexities inherent in activated carbon materials.

## 2 Properties and structural characteristics of activated carbon

Activated carbon is produced through a two-step process: carbonization and activation. The carbonization process involves the pyrolysis of organic precursors in an inert atmosphere at temperatures ranging from 373 to 1273 K, resulting in a carbon-rich char with low surface area Yang [56]*;* Bansal, Jean-Baptiste and Fritz [6]*;* Bandosz, Biggs, Gubbins, Hattori, Uyama, Kaneko, Pikunic and Thomson [5]. This char is then subjected to an activation phase, which can occur physically (using steam or CO_2_ at elevated temperatures) or chemically (using activating agents such as phosphoric acid or potassium hydroxide). This activation process significantly increases the surface area and develops micro- and mesopores, making activated carbon a highly effective adsorbent and catalyst Bandosz et al. [5]*.*

Despite its widespread use, the atomic-level structure of activated carbon remains poorly defined. This ambiguity has led to the development of several theoretical models to describe its properties. Some models adopt a traditional perspective, representing the microstructure as a twisted network of carbon atoms connected by various bonding groups. This model explains the material’s hardness and its resistance to graphitization, both characteristic features of non-graphitizing carbons. Other models draw parallels between microporous activated carbons and fullerene-like structures, emphasizing the role of curvature and hybridization in defining their unique properties Harris [20].

The structural complexity of activated carbon is also influenced by its synthesis methods and the choice of precursor materials. Variations in precursor composition, carbonization temperature, and activation conditions can lead to significant differences in porosity, surface area, and functional group distribution Yang [56]*;* Bansal et al. [6]. For instance, carbon derived from coconut shells typically exhibits a higher surface area compared to that derived from wood, which can affect its performance in adsorption applications.

## 3 Advances in experimental techniques

Since the early 1900s, researchers have conducted extensive experimental analyses to elucidate the structure and chemistry of activated carbons. Key analytical techniques include X-ray diffraction (XRD), electron diffraction, Raman spectroscopy, Fourier-transform infrared spectroscopy (FTIR), and high-resolution transmission electron microscopy (HRTEM) Bandosz et al. [5]. Each of these techniques offers unique insights into the atomic arrangement, phase composition, and surface functionalities of activated carbon materials.

XRD provides information on the crystalline structure and helps identify the presence of different carbon allotropes, while Raman spectroscopy can reveal details about the sp^2^ and sp^3^ hybridization states of carbon atoms, giving insights into the degree of disorder within the material. FTIR spectroscopy, on the other hand, is valuable for characterizing surface functional groups, which play a crucial role in the adsorption processes. HRTEM enables researchers to visualize the nanoscale structures of activated carbons directly, confirming the presence of various pore types and defect structures Bandosz et al. [5].

Moreover, advancements in computational modeling have complemented experimental findings, leading to the development of sophisticated theoretical models that account for the dynamic behavior of activated carbon under different conditions. For instance, molecular dynamics simulations and Monte Carlo methods allow for the prediction of adsorption isotherms, pore size distributions, and the interactions between activated carbon and various adsorbates. These computational techniques have become increasingly important for optimizing activated carbon properties for specific applications, providing a framework for tailoring materials to meet the demands of diverse industrial processes Harris [21].

In summary, the study of activated carbon modeling is a rapidly evolving field, driven by both experimental and theoretical advancements. Understanding the complex relationship between the nanoscale structure and macroscopic properties of activated carbon is critical for enhancing its performance in various applications. Continued research in this area will not only refine existing models but also pave the way for the design of innovative carbon-based materials with tailored functionalities, addressing the growing needs in environmental remediation, energy storage, and catalysis. By integrating experimental data with advanced computational techniques, we can unlock new insights into the behavior of activated carbons, ultimately contributing to the development of sustainable and efficient solutions in a range of industries.

## 4 Bonds in carbon structures

Regarding the types of bonding present in carbon structures, graphite has graphene layers with a certain degree of displacement. However, the presence of interlayer bonds is not evident, but van der Waals interactions between carbon atoms of different layers are observed at distances ranging from 0.153 nm to 0.132 nm.

The presence of sp^2^ hybrid orbitals with bond angles of 120° in graphite leads to the formation of *σ* bonds between the carbons in the graphitic network, and pure P_z_ orbitals allow for the formation of bonds. Considering the presence of *sp*^3^ hybridized carbon atoms with tetrahedral geometry in such structures subjected to high temperatures confers instability, specifically on tetrahedrally bonded carbons present in amorphous layers at temperatures above 630 °C. The presence of such hybridized carbons does not explain the resistance to graphitization Harris [20].

The structure of carbons shows a notable reduction in the degree of ordering compared to hexagonal graphite. This disorder in the structure leads to the formation of a multiscale porous structure, giving rise to structures such as porous carbons and vitreous carbons Marsh and Reinoso [32]. There are basically two types of carbons: the so-called graphitic carbons (anisotropic carbons), which are anisotropic carbons with stacked graphene layers and planarity, as shown in X-ray diffraction studies. The lines exhibited in the diffractograms obtained for this type of carbons tend to become sharper at temperatures above 2000 °C. On the other hand, the non-graphitizable carbons (isotropic carbons) do not exhibit X-ray diffraction lines, even at elevated temperatures. These carbons show a pronounced porosity Marsh and Reinoso [32].

Non-graphitizable carbons exhibit short-range order, which is not eliminated despite the thermal treatment they undergo for their production. However, a considerable increase in the treatment temperature leads to the appearance of isotropic zones and spaces between the graphenic fragments Pikunic, Pellenq, Thomson, Rouzaud, Levitz and Gubbins [46].

X-ray diffraction provides information about the bulk structure of carbon materials. Specifically, the typical diffrac- tograms for activated carbons indicate the presence of pores and non-uniform electron density structures, as well as signals associated with nanographitic unit structures of sizes between 1 and 3 nm. Data obtained from X-ray diffraction studies Franklin [17]*;* Biscoe and Warren [8] have confirmed that activated carbon consists of hybridized carbons. Additionally, spectroscopic studies have identified the presence of heteroatoms such as oxygen, nitrogen, and sulfur in the form of heterocyclic rings and functional groups on the surface of the material Bansal et al. [6].

However, the molecular structure of activated carbons was a subject of debate for years. It was not until the advent of high-resolution transmission electron microscopy (HRTEM) in the 1960s that a greater structural understanding was achieved. In this context, the work of Oberlin et al. Oberlin and Oberlin [41]*;* Oberlin [38]*;* Oberlin and Terriere [42]*;* Oberlin, Endo and Koyama [40]*;* Inagaki, Takeichi, Hishiyama and Oberlin [23]*;* Oberlin, Boulmier and Terriere [39] introduced terms like the basic structural unit (BSU) Oberlin and Oberlin [41], which is the fundamental unit for the construction of activated carbon. This unit consists of small aligned polyaromatic molecules that form layers, as named by Oberlin. The BSUs exhibit a lack of order due to the presence of functional groups, an interlayer spacing greater than that of graphite, and a low order in the c direction, known as turbostratic structure Biscoe and Warren [8]. Oberlin proposed that the BSUs assemble to form regions of local molecular orientation (LMO) Oberlin [38], which correspond to the second level of structural hierarchy in activated carbon. These regions then assemble again to form more complex structures.

Furthermore, Harris et al. Harris [20]*;* Harris and Tsang [22]*;* Harris, Burian and Duber [19] conducted HRTEM image studies of microporous carbons, finding that these are formed by curved graphene layers whose curvature is closely related to the random presence of non-hexagonal rings. The findings obtained by Harris et al. were consistent with the studies reported by Oberlin, where the nature of the rings in the BSUs and LMOs is not imposed. The local molecular orientation (LMO) considers structural parameters such as L_a_ and L_c_ associated with range and height parameters, considering the degree of order within the region and the interlayer spacing (*d*002). All these parameters largely depend on the nature and chemistry of the precursor, as well as the thermal treatment (carbonization) Oberlin [38].

The precursor plays a fundamental role in the structure of the final material, so it is necessary to understand the starting molecular structure. This involves modeling the structure and its evolution under relevant conditions. However, despite the limited structural understanding at the atomic and molecular level of the precursors and, consequently, the materials obtained, the use of molecular modeling in combination with analytical techniques has allowed significant advances in this understanding Bansal et al. [6].

Finally, the production process of these materials involves a carbonization stage, from which intermediate materials with specific characteristics, such as high carbon content, are obtained. Subsequently, an activation process is carried out to obtain the final material. In this activation process, two main methods stand out: physical activation and chemical activation Inagaki et al. [23].

## 5 Carbonization

Carbonaceous materials are obtained after a thermal treatment known as carbonization, where precursor materials undergo a significant increase in temperature in an inert atmosphere. This process involves profound changes in both the chemical composition and molecular structure of the material.

From a chemical perspective, the carbonization process fundamentally involves two stages. The primary stage involves the loss of aliphatic species and non-carbon species from the precursor, resulting in a highly aromatic carbon with fractions of oxygen, sulfur, nitrogen, and hydrogen Rodriguez-Reinoso [48]*;* Marsh and Reinoso [32]. This stage is followed by a secondary carbonization, where (-CH) groups attached to aromatic rings in the network are released as low molecular weight gases, and the remaining aromatic molecules are linked and cross-linked through C-C and C-O bonds. These stages explain the evolution and change in the microstructure of the material, where the release of bulky aliphatic molecules from between fragments of the aromatic network allows the mobilization of aromatic molecules, forming the basic structural units (BSUs). These disoriented BSUs can generate nanoscale regions of local molecular order (LMO), which can grow, organize, and even merge with neighboring regions Bansal et al. [6].

The carbonization process transforms the precursor into a three-dimensional network, a macroatomic system of carbon atoms, where the elimination of species such as H_2_O, CO_2_, free radicals, and heteroatoms creates a stable intermediate with a high carbon content. The heating conditions lead to a rearrangement that results in the development of nanometric spaces. Once this process is complete, a discontinuous solid with a network of pores is obtained Marsh and Reinoso [32].

During the carbonization process, the microporous structure of the carbons is developed. However, the atomic-level mechanism is unknown, and it is not possible to understand why graphitizable or non-graphitizable carbons are formed. It is known that the characteristics of the precursors are fundamental. Non-graphitizing carbons are formed from substances with a lower proportion of hydrogen and a higher proportion of oxygen atoms compared to materials that form graphitizable carbons. The materials that lead to graphitizable carbons exhibit physical properties such as forming liquid phases at temperatures between 400 and 500 °C, which generates the necessary mobility to form preferentially oriented regions. In contrast, precursors that lead to non-graphitizable carbons remain solid during thermal treatments without melting Rodriguez-Reinoso [48]*;* Marsh and Reinoso [32]*;* Harris [21].

The mobility generated in the BSUs largely depends on the aliphatic phase present in the precursor, the rate of elimination, the process temperature, and the cross-linking between aromatic molecules, where heteroatoms like oxygen and sulfur facilitate this cross-linking. Studies have found that the production of activated carbons does not require secondary carbonization; however, the presence of a precursor with high oxygen content is necessary for their manufacture Bansal et al. [6].

Among the various techniques used for structural studies is electron spin resonance (ESR), which measures the concentrations of free radicals in a material and the properties of free electrons, providing information about the change in surface chemical composition as a function of temperature Marsh and Reinoso [32]. ESR studies by Mrozowski have reported that free spin concentrations are significantly reduced in graphitizable carbons compared to non-graphitizable ones, where larger graphene sheets (present in graphitizable carbonaceous materials) facilitate electron pairing within the layers. However, the low propensity for electron pairing in the sheets of non-graphitizable carbons indicates the absence of long graphene sheets Harris [20].

The maximum free spin concentration varies with temperature due to structural variation and the chemical composition of defective micrographene sheets. Moreover, the formation of graphitizable carbon is not porous; to form porous carbon, reactions are required that force the separation of stacked graphene layers Marsh and Reinoso [32].

## 6 Models of carbon structures

Given the chemical, structural, and porosity variability of carbons at multiple scales, understanding their precise atomic and molecular structure has remained a challenge, even after decades of experimental research. Numerous models have been proposed to explain their physical and chemical behaviors, but each has its limitations. These models, based on experimental data such as X-ray diffraction, electron microscopy, and other spectroscopic techniques, aim to elucidate the unique characteristics of both graphitizing and non-graphitizing carbons, which exhibit significantly different behaviors under high-temperature treatment and mechanical stress.

Over the years, several microstructural models have been suggested to describe the architecture of these materials at the nanometer scale. The most influential early models, including Rosalind Franklin’s model and the ribbon model proposed by Crawford-Johnson, have become the foundation for understanding the disordered and often non- graphitizing nature of certain carbons. These models differ significantly in their treatment of atomic arrangement, defect structures, and bonding characteristics.

### 6.1 Rosalind Franklin’s model

**Fig. 1:**
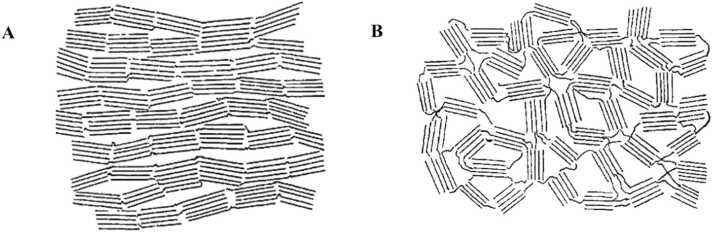
Franklin’s representations of A graphitizing and B non-graphitizing carbons Franklin [17].

**Fig. 2:**
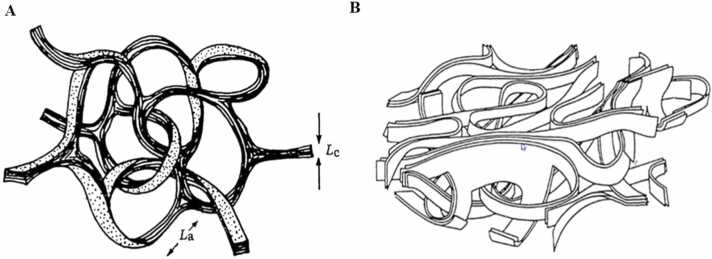
A. Jenkins and Kawamura’s model for the structure of glassy carbon Jenkins et al. [24] and B. Ban’s model for PVDC carbon heat-treated at 1950°C Ban et al. [4].

**Fig. 3:**
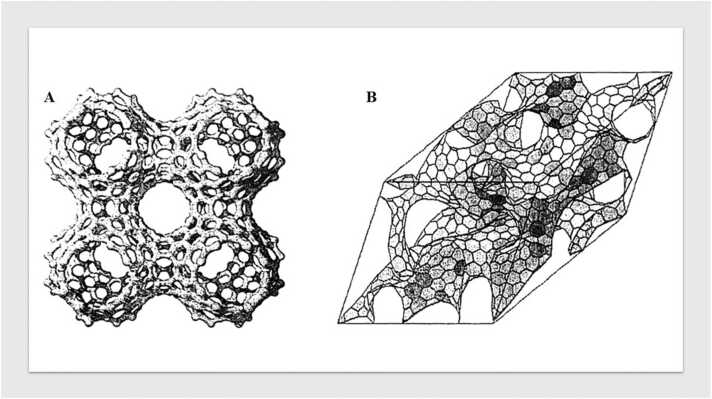
A. Periodic graphitic structure according to Terrones et al. Mackay and Terrones [31]; Terrones and Terrones [50] and B. ’Schwartzite’ type structure proposed by Townsend et al. Townsend et al. [53].

**Fig. 4:**
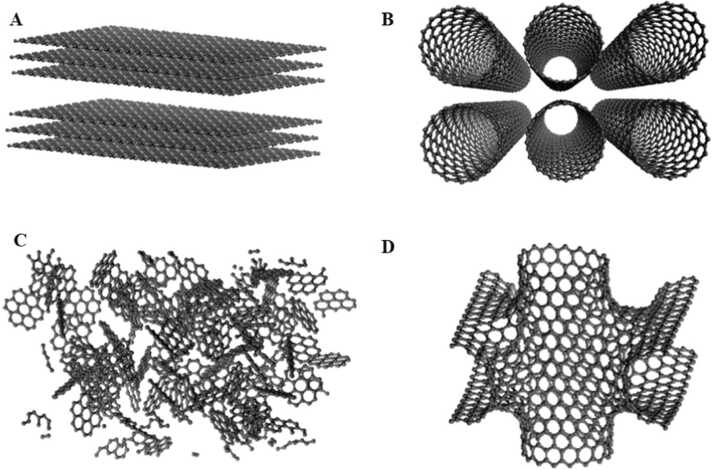
Models of nanoporous structures considered by Kumar et al. A. slit pore; B. nanotube; C. random structure; and D. foam Kumar et al. [26].

**Fig. 5:**
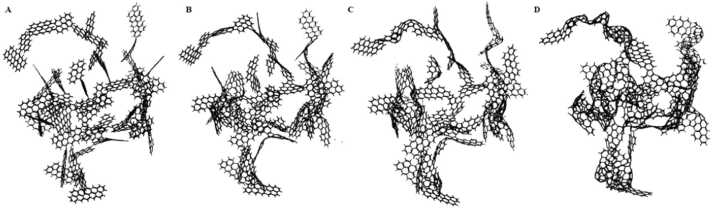
Structural model evolution of the carbon nanostructure as a function of the reduction in the H/C ratio (therefore, due to the increase in temperature) reported by Acharya et al. Acharya et al. [1].

**Fig. 6:**
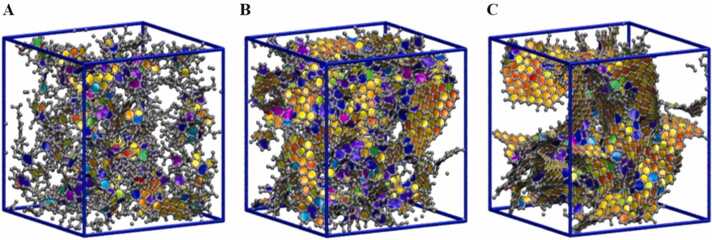
Models reported by Gubbins et al. for disordered nanoporous carbon (DNC) structures obtained through QMD simulations at different relative quench rates of A 32, B 8, and C 1 Palmer and Gubbins [44].

**Fig. 7:**
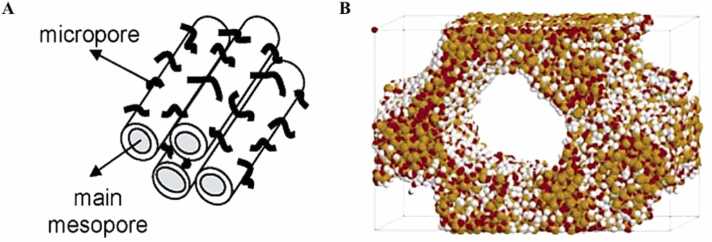
Atomistic structure of SBA-15 proposed by Gubbins et al.: A schematic showing the meso- and micropores; B snapshot of the simulation box (yellow, silicon; red, oxygen; white, hydrogen) Bhattacharya and Gubbins [7].

One of the earliest and most prominent models is attributed to Rosalind Franklin’s groundbreaking research in 1951. Franklin proposed that both graphitic and non-graphitic carbons consist of small crystalline graphite domains, made up of nanoscale graphite units or layers. She argued that the stacking of these layers was governed not by the movement of individual carbon atoms but by the motion of these entire layers or fragments. This layer movement is more pronounced in graphitizable carbons, where weak van der Waals interactions allow for the parallel stacking of the sheets. In non- graphitizable carbons, Franklin observed a more chaotic orientation of these units, with strong inter-unit bonds that resist reordering even under high temperatures, thus preventing the formation of well-ordered graphitic structures. This model successfully explained why graphitic carbons undergo ordered transformation at elevated temperatures, while non-graphitic carbons resist graphitization even at temperatures exceeding 3000 °C. Franklin’s contributions provided one of the earliest frameworks for understanding the atomic-scale properties of carbon, laying the groundwork for subsequent models and experimental explorations Rodriguez-Reinoso [48]*;* Marsh and Reinoso [32]*;* Franklin [17] (Fig. 1, Fig. 2, Fig. 3, Fig. 4, Fig. 5, Fig. 6, Fig. 7).

Franklin’s work also demonstrated the importance of precursor materials in determining carbon structure. She identified that highly porous carbons with a substantial internal surface area could be produced through the pyrolysis of organic materials such as polyvinylidene chloride (PVDC) and sucrose. These observations highlighted the critical role of precursor chemistry and processing conditions in influencing the final carbon material’s structural characteristics, including its porosity, surface area, and degree of graphitization Franklin [17].

### 6.2 The ribbon model, vitreous carbon, and other models

In addition to Franklin’s model, the ribbon model developed by Ban et al. Ban, Crawford and Marsh [4] and the vitreous carbon model proposed by Jenkins et al. offer alternative explanations for non-graphitizing carbons. Ban’s ribbon model is based on the observation that these materials are composed of curved graphite-like sheets, where the carbon atoms primarily exhibit sp^2^ hybridization, forming hexagonal ring structures. This model emphasizes the curvature and randomness of these graphene-like sheets, which prevents the formation of a fully crystalline structure. Jenkins’ vitreous carbon model further elaborates on this concept Jenkins, Kawamura and Ban [24], suggesting that non-graphitizing carbons possess an amorphous glass-like structure, where the disordered arrangement of *sp*^2^ hybridized carbon atoms leads to high hardness and resistance to graphitization, similar to what is observed in glassy materials.

Ergun et al. Ergun and Alexander [13]*;* Ergun and Tiensuu [14] later introduced an alternative approach by proposing that sp^3^ hybridization may also occur in some carbon materials. Their X-ray diffraction studies indicated the presence of diamond-like domains in non-graphitizing carbons, which could explain the materials’ high hardness and their inability to transform into graphite. These diamond-like domains, although not prevalent, were hypothesized to contribute to stability and resistance to graphitization. However, the presence of significant sp^3^ hybridization remains a matter of debate, as these tetrahedrally bonded carbon atoms are generally unstable at high temperatures, particularly in amorphous layers above 630 °C.

Despite the wide variety of models proposed, many failed to account for the strong cross-linking required to maintain the stability of carbon structures at temperatures exceeding 3000 °C Rodriguez-Reinoso [48]*;* Marsh and Reinoso [32]. Additionally, the flexibility of graphite sheets at such high temperatures posed challenges in explaining the non-graphitizable nature of some carbons. Ergun’s studies with X-ray diffraction showed that these patterns could not conclusively eliminate the possibility of *sp*^3^ hybridized carbons Ergun and Alexander [13]*;* Ergun and Tiensuu [14]. Meanwhile, Ruland’s work complicated the matter by showing the 0002 line of graphite in non-graphitizing carbons, which was difficult to reconcile with the proposed presence of *sp*^3^ carbons.

### 6.3 Further advances in carbon structure models

Oberlin’s Oberlin and Oberlin [41]*;* Oberlin [38]*;* Oberlin and Terriere [42]*;* Oberlin et al. [40]*;* Inagaki et al. [23]*;* Oberlin et al. [39] research contributed significantly to resolving some of these uncertainties by leveraging high-resolution transmission electron microscopy (HRTEM) to visualize the molecular arrangement in carbon materials. His studies showed that, for graphitizable carbons, the 0002 fringes corresponding to the graphene layers were clearly visible, appearing as ribbon-like structures. Oberlin’s work suggested that while the interpretation of HRTEM images could be subjective, they provided crucial insights into the fundamental differences between graphitizable and non-graphitizable carbons. Specifically, his findings emphasized the importance of molecular flexibility and the presence of defects, which play a critical role in determining the carbon’s response to high- temperature treatments and mechanical stress.

In conclusion, the variety of models developed over the years reflects the complex nature of carbon materials, which can exhibit both graphitic and non-graphitic characteristics depending on their precursor chemistry, processing conditions, and thermal history. The understanding of these models continues to evolve with advances in experimental techniques, particularly in microscopy and spectroscopy, offering new insights into the atomic and molecular architecture of these versatile materials.

## 7 Activation process and surface area development

In the development of carbon structure models, the activation process plays a crucial role in the formation and enhancement of surface area in graphitic and non-graphitic carbons. This process is primarily driven by two key mechanisms: the oxidation or burning of carbon fragments within microporous regions, which significantly expands the material’s surface area, and the selective attack on pentagonal rings, a process that induces openings within the laminar structure. This, in turn, creates vacancies and significantly increases porosity, facilitating greater accessibility for adsorbates and other molecules Marsh and Reinoso [32].

Moreover, it is essential to recognize that amorphous carbons do not conform strictly to six-membered ring configurations, as initially believed. *Byrne and Marsh* Patrick [45], in their 1995 study, demonstrated that carbons generated through pyrolysis of cellulosic materials contain a mixture of small *sp*^2^ carbon clusters and *sp*^3^ hybridized carbons, often forming non-hexagonal ring structures (five- to seven-membered rings). This structural variety results in a complex matrix that exhibits both graphitic and non-graphitic characteristics, influencing their adsorption and catalytic properties.

The introduction of the ’Schwarzite’ model, which incorporates curvature in the carbon sheets due to the presence of non-hexagonal rings, particularly seven-membered ones, has been instrumental in advancing the understanding of these materials. Schwarzite structures present continuous, seamless surfaces with highly porous architectures, with pore diameters typically ranging from 0.5 to 1 nm. These models, although hypothetical, provide valuable insights into how curvature and defects within the carbon framework impact the material’s physical and chemical properties Mackay and Terrones [31]*;* Lenosky, Gonze, Teter and Elser [27]*;* Terrones and Terrones [50]*;* Townsend, Lenosky, Muller, Nichols and Elser [53].

Recent advancements in high-resolution transmission electron microscopy (HRTEM) have further refined these models. Studies have revealed highly disordered carbon structures, particularly those obtained via the pyrolysis of polyvinylidene chloride (PVDC) and sucrose at 700 °C in an inert atmosphere. These carbons display individual, rolled graphene layers, which, upon further thermal treatment at 2600 °C, transform into closed nanoparticles with distinct saddle points, a characteristic directly linked to the presence of seven-membered rings Harris [20]. This transformation highlights the dynamic nature of carbon materials under varying thermal conditions and suggests potential applications in fields requiring materials with specific structural attributes, such as catalysis and energy storage.

## 8 Recent models and microstructures

In more recent models of non-graphitizable carbons, a shift has been made towards microstructures that resemble fullerene-like sealed capsules. These structures are characterized by gas-impermeability and exhibit a high degree of curvature. However, these models, while helpful, tend to overlook the role of small graphitic fragments, which contribute to the overall reactivity and oxidation behavior of non-graphitizable carbons. This oversight underscores the importance of continued refinement of models to more accurately reflect the real-world behavior of these materials under various conditions Marsh and Reinoso [32]*.*

Harris’s model Harris [20] represents a more nuanced understanding of non-graphitizable carbons, incorporating discrete, randomly arranged fragments of curved graphene sheets with pentagonal ring defects. This model not only explains the interconversion of these sheets into closed carbon nanoparticles under thermal treatment but also incorporates the influence of precursor material structure on the final carbon morphology. The presence of pentagonal rings, retained from the precursor, introduces curvature and enhances microporosity, with pore sizes typically in the range of 0.5–1 nm. This random distribution of structural defects contributes to the material’s low density and high surface area, a characteristic reminiscent of fullerene soot.

Further exploration of these models suggests that non-graphitizable carbons are highly resistant to graphitization, even at extreme temperatures, and instead tend to form a porous, amorphous structure. The discovery of carbon nanotubes, fullerenes, and other nanostructures has reinforced the idea that non-hexagonal rings can stabilize within an sp^2^ carbon framework, allowing for the formation of unique microstructures with promising applications in nanotechnology and materials science Marsh and Reinoso [32]*.*

Advanced techniques such as transmission electron microscopy (TEM) have enabled a clearer distinction between graphitizable and non-graphitizable carbons. The former exhibits small, aligned, and well-packed layers, while the latter displays a disordered, isotropic structure with spiraled or curved graphene layers. Upon thermal treatment, graphitizable carbons develop into highly ordered structures composed predominantly of graphite crystals, whereas non-graphitizable carbons form porous materials with little long-range crystalline order. The curved and faceted graphene layers of non-graphitizable carbons, typically 1–2 nm thick and 5–15 nm in length, give rise to randomly distributed pores, contributing to their unique adsorption properties Rodriguez-Reinoso [48]*;* Marsh and Reinoso [32]*;* Harris [21]*.*

Peter H. J.’s more recent models introduce the concept of microporous carbons containing discrete fragments of curved graphene sheets with a high density of non-hexagonal rings, including both pentagons and heptagons. This model aligns with observed microporosity in fullerene soot and reflects a similar porosity range of 0.5–1 nm. TEM analysis further supports this model by revealing a randomly distributed network of pentagons and heptagons embedded within a hexagonal carbon framework, highlighting the complexity of these structures and their potential for tailored applications in gas storage, catalysis, and filtration Harris et al. [19]*;* Tsang, Harris, Claridge and Green [54]*;* Bursill and Bourgeois [11]*;* Bourgeois and Bursill [10].

### 8.1 Current models and simulation techniques: GCMC and MD

The field of porous materials, particularly activated carbons, has witnessed significant advancements in recent years, primarily driven by improvements in computational hardware and sophisticated simulation techniques. Alongside the evolution of experimental data from various analytical methods, these advancements have collectively contributed to a much more refined understanding of the phenomena associated with porous carbons. This includes the modeling of pore formation, structural evolution, adsorption properties, and the behavior of these materials under various conditions.

The application of simulation techniques such as Grand Canonical Monte Carlo (GCMC) and Molecular Dynamics (MD) has been central to these developments, offering powerful tools to probe the intricate details of material structures and their interactions with gases, liquids, and other adsorbates at the molecular level.

### 8.2 The role of computational techniques in model development

Historically, the study of porous carbon materials relied heavily on empirical data and physical experimentation. While these approaches provided valuable insights into the general properties of activated carbons—such as surface area, pore size distribution, and adsorption capacities—they were limited in their ability to predict or explain the molecular-scale phenomena that govern these properties. The introduction of advanced computational techniques has fundamentally transformed this landscape, allowing researchers to model the atomic and molecular interactions within carbon materials with unprecedented precision. Techniques like GCMC and MD, along with hybrid approaches such as Hybrid Reverse Monte Carlo (HRMC) and Quench Molecular Dynamics (QMD), have enabled the detailed prediction of material behavior, going beyond what experimental methods alone could achieve.

#### 8.2.1 Grand canonical Monte Carlo (GCMC) simulations in carbon modeling

One of the primary methods utilized in the study of porous materials is the Grand Canonical Monte Carlo (GCMC) simulation. GCMC is particularly well-suited for modeling adsorption phenomena because it allows the number of particles, volume, and temperature to fluctuate in the simulation environment, closely mimicking real-world adsorption processes where gas molecules enter and exit the pores of the material.

*Kumar et al*. Kumar, Salih, Lu, Müller and Rodríguez-Reinoso [26] made substantial progress by applying GCMC simulations to model the transformation of polymeric precursor structures into porous carbon. Their study focused on polyfurfuryl alcohol, a precursor commonly used in the synthesis of non-graphitizable carbons. The initial modeled structure began with a predefined density of 1.72 g/*cm*^3^, which is consistent with experimental values. The resulting model exhibited a hexagonal network interspersed with a 10–15 % distribution of non-hexagonal rings, primarily five- and seven-membered rings, which is consistent with the literature data on carbon materials. These simulations provided new insights into the atomic-level transformations that occur during carbonization, particularly the formation of pores and the evolution of structural defects that contribute to the material’s porosity and surface area.

In addition to Kumar et al.’s work, Acharya et al. Acharya, Strano, Mathews, Billinge, Petkov, Subramoney and Foley [1] used GCMC to explore the structural evolution of microporous carbon during thermal treatment. Their simulations began with fragments of hexagonal ring structures, terminated by hydrogen atoms, representing the early stages of carbonization. As the temperature increased, the model predicted the formation of non-hexagonal rings within the carbon network, specifically the introduction of five- and seven-membered rings, which are crucial for the development of microporosity. This work provided further evidence that the evolution of pore structures in carbon materials is closely linked to the thermal history and the specific atomic configurations present in the precursor material.

Bahamon et al. Bahamon, Carro, Guri and Vega [3] implemented an extension of the model proposed by Segarra and Glandt Segarra and Glandt [49], in which the carbon-based material is represented as a disordered matrix of functionalized curved graphene sheets. In their study, graphene planes are modeled as platelets incorporating polar functional groups and structural defects—features not explicitly considered in the original Segarra and Glandt model. The approach introduced by Bahamon et al. presents conceptual similarities with the model developed by Liu and Monson Liu and Monson [30]*.* To construct the system, between 10 and 20 percent of the atoms in the inner carbon layers were removed in order to enhance surface roughness. This modification enabled the development of a qualitatively accurate model capable of providing meaningful insights into the physisorption mechanisms in such materials as a function of structural parameter variations.

On the other hand, Shi et al. Li, Song, Zhao, Rugarabamu, Diao and Gu [29] employed structural models of activated carbons featuring diverse pore size distributions and functional groups. These models were developed as an extension of the structural packing approach proposed by Segarra and Glandt Segarra and Glandt [49]. Their construction involved a random arrangement of non-hexagonal rings, along with a controlled distribution of pore shapes and sizes. The incorporation of functional groups—such as hydroxyl, carboxyl, phenolic, and pyridinic moieties Gelb and Gubbins [18]*;* Boehm [9]—together with structural defects introduced via the inclusion of approximately 15 percent pentagonal and heptagonal rings Harris [21]*;* Leyssale, Da Costa, Germain, Weisbecker and Vignoles [28]*;* Morris, Contescu, Chisholm, Cooper, Guo, He, Ihm, Mamontov, Melnichenko, Olsen et al. [35]*;* Kumar, Lobo and Wagner [25], enabled the investigation of the physicochemical mechanisms governing benzene adsorption in micro- and mesoporous environments under varying temperature conditions.

#### 8.2.2 Hybrid reverse Monte Carlo (HRMC) simulations and their applications

While GCMC has been invaluable in modeling adsorption phenomena and structural evolution, Hybrid Reverse Monte Carlo (HRMC) simulations offer a complementary approach that excels in constructing highly random, disordered nanoporous carbon models. Palmer and Gubbins Palmer and Gubbins [44] utilized HRMC simulations to develop a model of nanoporous activated carbon, which enabled them to predict the hydrogen adsorption properties of the material with remarkable accuracy. By combining experimental data with simulation results, they were able to fine-tune their models, leading to a deeper understanding of how pore size distribution and surface chemistry influence adsorption behavior.

The HRMC approach is particularly useful for modeling materials like activated carbons, which often exhibit significant structural disorder and heterogeneity. These materials do not conform to a single, well-defined crystalline structure but instead consist of a complex mix of amorphous and semi-crystalline regions, making them difficult to model using traditional crystallographic techniques. The HRMC method allows for the incorporation of this disorder directly into the simulation, producing models that more closely resemble the real-world structures of nanoporous carbons.

In 2008, Thanh et al. Nguyen, Cohaut, Bae and Bhatia [37] proposed a constrained hybrid reverse Monte Carlo (HRMC) methodology for the atomistic modeling of the microstructure of activated carbons. The approach is based on an initial configuration derived from experimental characterization data—specifically, pore size distributions (PSD) and wall thicknesses—obtained through the interpretation of argon adsorption isotherms at 87 K. To this end, an enhanced version of the slit-pore model incorporating a finite wall thickness (FWT) Nguyen and Bhatia [36] was employed, which effectively addressed the limitations of conventional HRMC methods, particularly in describing short-range interaction potentials. This modeling procedure is especially well-suited for representing anisotropic carbon materials, such as activated carbon fibers.

#### 8.2.3 Quench molecular dynamics (QMD) and reactive state sum potentials (RSS)

In addition to Monte Carlo methods, Molecular Dynamics (MD) simulations have played a crucial role in understanding the dynamic behavior of carbon materials. *Shi* Palmer, Llobet, Yeon, Fischer, Shi, Gogotsi and Gubbins [43] combined Quench Molecular Dynamics (QMD) with reactive state sum (RSS) potentials to model the porous nanostructures of activated carbons. This approach allowed them to simulate the ring size distribution, pore size distribution, and angular distribution of bonds within the carbon network. By incorporating the influence of cooling rates during thermal treatment, Shi’s work provided new insights into how the rate of carbonization affects the final structure of the material. Faster cooling rates tended to produce more disordered structures with smaller, more irregular pores, while slower cooling rates allowed for the formation of larger, more well-defined pores.

Yang et al. Yang, Ju and Huang [55] expanded upon this work by implementing a structural prediction method to enhance the QMD approach. They incorporated time-marked force bias (tfMC) into their simulations, which allowed them to create larger, more realistic models of activated carbons. Their simulations used empirical bond order potentials (REBD) to describe bond formation and breaking events within the carbon network. These potentials took into account nucleus-nucleus repulsive interactions, electron-electron repulsive interactions, and attractive nucleus- electron interactions, all of which depend on the distance between atoms, the types of neighboring atoms, and the bending angles between bonds. This advanced approach yielded predictions for local hybridization states, coordination numbers, and surface areas that were consistent with experimentally measured values for commercial activated carbons, which typically range from 500 to 1500 *m*^2^/g Pré, Huchet, Jeulin, Rouzaud, Sennour and Thorel [47]*;* Baghel, Singh, Prasad, Pandey and Gutch [2]*.*

Thompson et al. Thompson, Dyatkin, Wang, Turner, Sang, Unocic, Iacovella, Gogotsi, Van Duin and Cummings [52] implemented this methodology to generate atomistic models of carbide-derived carbons (CDCs), which exhibit nanoporous architectures with high specific surface areas, tunable porosity, and variable degrees of graphitization depending on the synthesis method. The construction of these models involved the use of reactive force fields in combination with post-quenching compression steps, resulting in a substantial improvement over models generated solely via quenched molecular dynamics (QMD). Notably, the enhanced models demonstrated improved representation of local bonding environments, more accurate pore size distributions, and qualitative morphological features consistent with those observed experimentally through aberration-corrected scanning transmission electron microscopy (STEM).

## 9 Pore size distribution and structural analysis techniques

Pore size distribution (PSD) is one of the most important properties of activated carbons, as it directly affects the material’s adsorption capacity and selectivity. To predict pore size distributions, Yang et al. Yang et al. [55] used Bhattacharya’s PSDsolv methodology Bhattacharya and Gubbins [7], a technique that integrates molecular dynamics simulations with experimental data to provide a detailed picture of the pore structure. This method allowed them to calculate the distribution of pore sizes across a wide range, from micropores (< 2 nm) to mesopores (2–50 nm) and macropores (> 50 nm). The resulting pore size distributions were consistent with those observed in experimental studies, confirming the validity of their model.

The combination of simulation techniques, such as GCMC, MD, and HRMC, with advanced structural analysis methods like PSDsolv, has allowed researchers to build highly accurate models of porous carbon materials. These models not only provide insights into the material’s structure but also enable the prediction of key properties such as surface area, pore size distribution, and adsorption capacity, all of which are critical for the design and optimization of carbon-based materials for applications in gas storage, catalysis, and filtration.

### 9.1 Incorporating new approaches for improved accuracy

Recent developments in computational techniques have further enhanced the accuracy and predictive power of these models. Mees Mees, Pourtois, Neyts, Thijsse and Stesmans [34] proposed a novel Monte Carlo methodology that incorporates time-marked force bias (tfMC) to predict the formation of nanostructures and phase transformations in carbon materials. This approach has been shown to provide more accurate predictions of the dynamic behavior of carbon atoms during thermal treatment, leading to more realistic models of the structural evolution of porous carbons.

Additionally, the integration of machine learning techniques with traditional simulation methods has opened up new possibilities for model refinement. Machine learning algorithms can be trained on experimental data to identify patterns and correlations that might not be immediately apparent from the simulation results alone. This allows for the continuous improvement of the models as new experimental data becomes available, leading to increasingly accurate predictions of material properties and behavior.

## 10 Conclusions

In conclusion, the integration of advanced computational techniques—such as Grand Canonical Monte Carlo (GCMC), Molecular Dynamics (MD), and Hybrid Reverse Monte Carlo (HRMC)—with high-precision experimental data has fundamentally transformed the understanding of activated carbon, enabling access to atomistic mechanisms that were previously unobservable. Next-generation models, particularly those based on ReaxFF and Quenched Molecular Dynamics (QMD), have demonstrated that microporosity is governed not only by the pore size distribution, but also by the density of topological defects (e.g., pentagonal and heptagonal rings) and the curvature of graphene sheets. For instance, recent simulations have shown that incorporating approximately 15 % of non-hexagonal rings can increase methane adsorption energy by up to 20 %, opening new pathways for the rational optimization of materials for gas storage applications.

These advances have immediate practical implications in several strategic domains:

-Rational materials design: Models based on negatively curved carbon structures, such as Schwarzites, predict specific surface areas of up to 3000 m²/g, offering promising prospects for the development of high-energy-density supercapacitors.

-Environmental sustainability: Simulations of the adsorption of emerging contaminants (e.g., PFAS and microplastics) on activated carbons functionalized with –COOH or –NH₂ groups indicate adsorption efficiencies exceeding 90 %, significantly reducing the need for extensive experimental trials.

-Clean energy: Models of carbide-derived carbons (CDCs) have guided the synthesis of sub-nanometer porous materials (<1 nm), optimized for hydrogen storage at 77 K, with gravimetric capacities of up to 5 wt%.

Nonetheless, critical challenges remain. These include the limited time scales accessible by current simulation methods, which hinder the study of slow chemical activation processes, and the need for experimental validation of multiscale models using advanced characterization techniques such as atom probe tomography (APT) or neutron scattering. To overcome these limitations, future research should prioritize the integration of machine learning techniques, training algorithms with data derived from high-resolution transmission electron microscopy (HRTEM) and GCMC simulations to enable accurate prediction of macroscopic properties from nanostructural features.

## CRediT authorship contribution statement

**Giraldo Liliana:** Writing – review & editing, Writing – original draft, Validation, Project administration, Methodology, Investigation, Formal analysis, Data curation, Conceptualization. **Moreno-Pirajan Juan Carlos:** Writing – review & editing, Writing – original draft, Validation, Methodology, Investigation, Funding acquisition, Formal analysis, Data curation, Conceptualization. **Larrota-Beltrán José I.:** Writing – original draft, Software, Methodology, Investigation, Formal analysis, Conceptualization.

## Declaration of Competing Interest

The authors declare that they have no known competing financial interests or personal relationships that could have appeared to influence the work reported in this paper. Furthermore, no funding agencies, organizations, or individuals had any involvement in the study design, data collection, analysis, interpretation, or writing of this manuscript.
